# A Case Report of Metallosis With a Failed Distal Femur Plate

**DOI:** 10.7759/cureus.10361

**Published:** 2020-09-10

**Authors:** Akshay Kumar, Syed Muhammad Hussain Zaidi, Badaruddin Sahito, Dileep Kumar, Maratib Ali

**Affiliations:** 1 Internal Medicine, Civil Hospital Karachi, Dow University of Health Sciences, Karachi, PAK; 2 Orthopaedic Surgery, Civil Hospital Karachi, Dow University of Health Sciences, Karachi, PAK

**Keywords:** metallosis, distal femur locking plate, distal femur nonunion

## Abstract

Metallosis is a very rare condition that poses a diagnostic challenge. Its precise incidence is not known and the condition should be suspected in patients who present with the clinical signs and symptoms discussed below. In particular, physicians and surgeons should pay special attention to whether metal prosthesis has been used for fracture repair or joint function. Here we report the case of a 40-year-old male who presented at an orthopedic surgery outpatient department in a tertiary care hospital with pain in the right distal thigh that had been ongoing for three months and swelling that had been ongoing for two months. According to the patient, he had received an operation for a right-sided distal femur fracture that was fixed with plate at a different hospital one year prior. Despite receiving all the appropriate physical exams and labs, and getting a radiologic workup, the diagnosis was unclear, and ultimately surgical exploration was conducted, which led to the diagnosis of metallosis. Although metallosis is a well-known complication, due to its rarity, physicians and surgeons often forget to keep metallosis as a differential that leads to diagnostic difficulties.

## Introduction

Metallosis is an extremely rare problem with unknown incidence [1] in which there is aberrant contact between metallic surfaces and metallic debris is released due to continuous abrasion [2]. Slowly, the debris invades the periprosthetic soft tissue and bone resulting in various symptoms [3], including small asymptomatic soft tissue lesions to significant osteolysis, necrosis, effusion, and bumps which can cause secondary pathological effects [4]. Patients presenting with these symptoms should be encouraged to return for clinical and radiographic follow-ups, and providers should consider performing a systematic evaluation [5]. Although metallosis is most commonly associated with arthroplasty of the hip, knee, and shoulder [2], here we report a case of a failed distal femur plate with a non-union femur with metallosis, which was subsequently treated with debridement and a distal femur locking plate.

## Case presentation

A 40-year-old male presented at an orthopedic surgery outpatient department in a tertiary care hospital with pain in the right distal thigh that had been ongoing for three months and swelling that had been ongoing for two months. According to the patient, he had received an operation for a right-sided distal femur fracture that was fixed with a plate at a different hospital one year prior. He explained that he had a fall six months prior without any acute symptoms. His pain had started three months after the fall, and it was mild to moderate, dull, continuous, aggravated by any leg movement, and relieved with analgesics. The pain was associated with swelling of the right distal thigh and around the knee joint. The swelling was initially mild but it got progressively worse. The patient denied any history of fever or weight loss. The patient’s history beyond what has been mentioned was insignificant.

**Figure 1:**
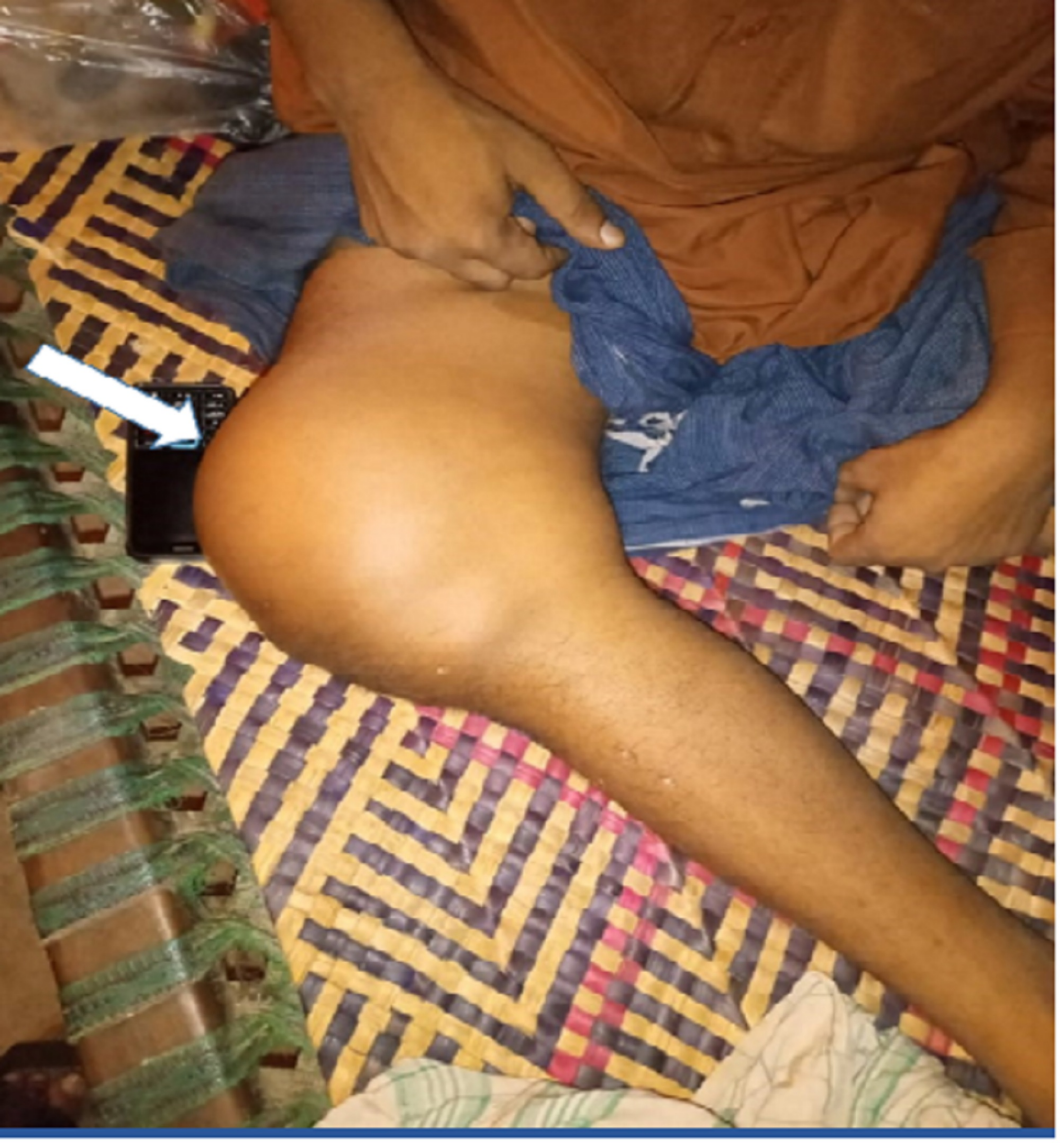
Examination showing the swelling around the right distal femur (white arrow) and around the knee joint measuring 10 x 15 cm in size.

A thorough physical examination revealed swelling around the distal thigh with the right leg placed in flexion; the overlying skin was normal with visible veins (Figure 1). The swollen area was non-tender, fluctuant, and measured 10 × 15 cm in size.

**Figure 2:**
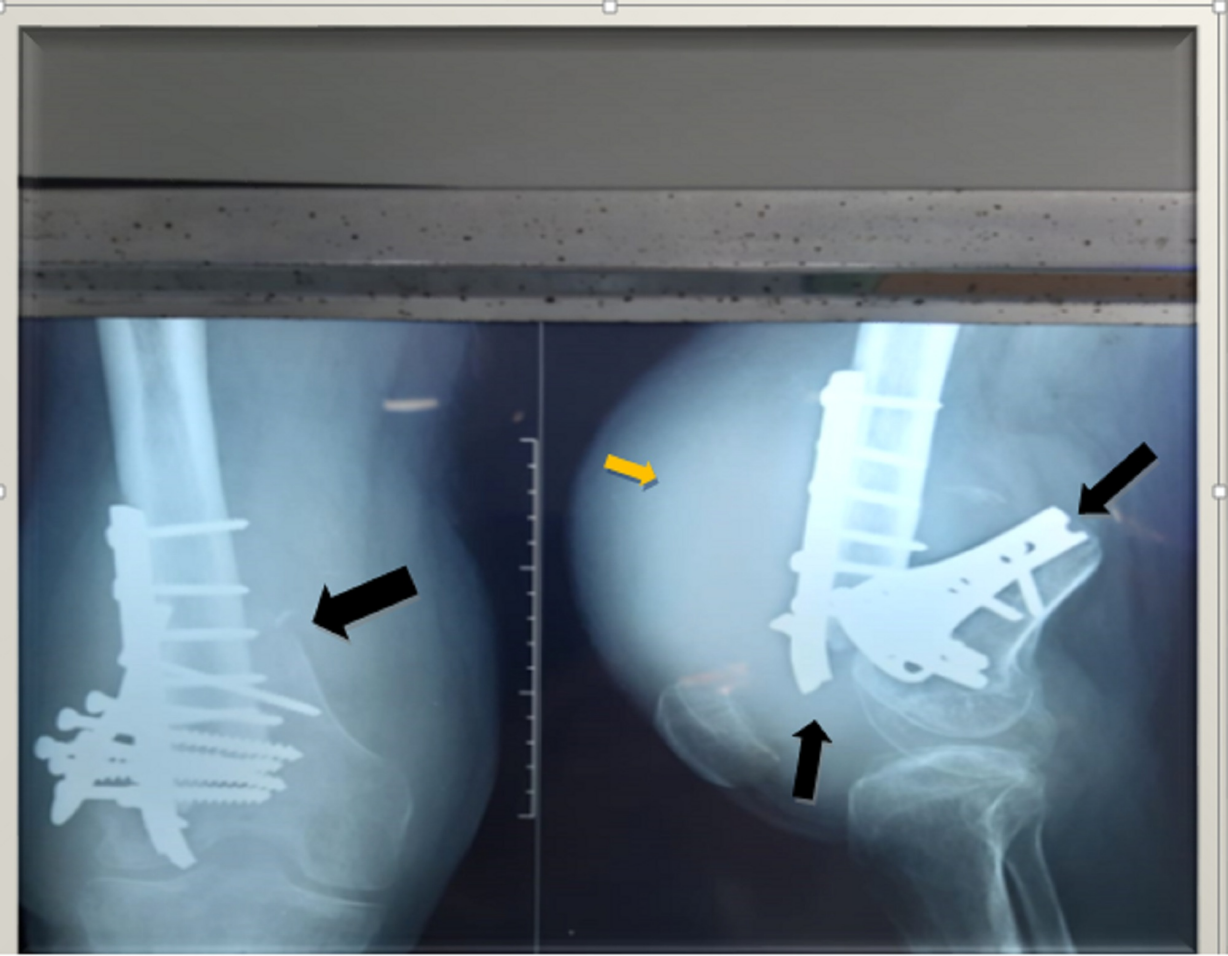
X-ray on the right shows the right distal femur in AP view, revealing implant breakage with a fracture of the femur (black arrow). The image on the left shows a lateral view of the right-sided distal femur, clearly revealing an implant breakage (black arrows) along with soft tissue swelling (yellow arrow). AP, anteroposterior

The patient’s blood workup came back normal. Radiographs were done, which showed a failed plate (distal femur condylar plate) and breakage of the implant (Figure 2). A diagnosis was made of implant failure with non-union secondary to a distal femoral fracture of the right side. The patient was then told about his condition and was thoroughly counseled regarding the need for surgical exploration, to which he consented.

**Figure 3:**
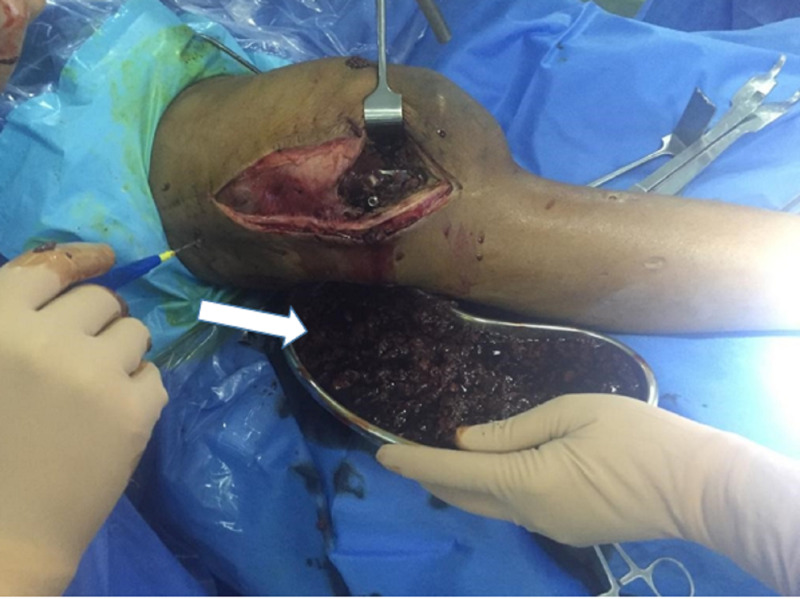
A lateral incision approach to the distal femur. Upon incision, there was drainage of turbid material (white arrow), which confirmed the diagnosis of primary metallosis (even though no metal on metal movement was present).

**Figure 4:**
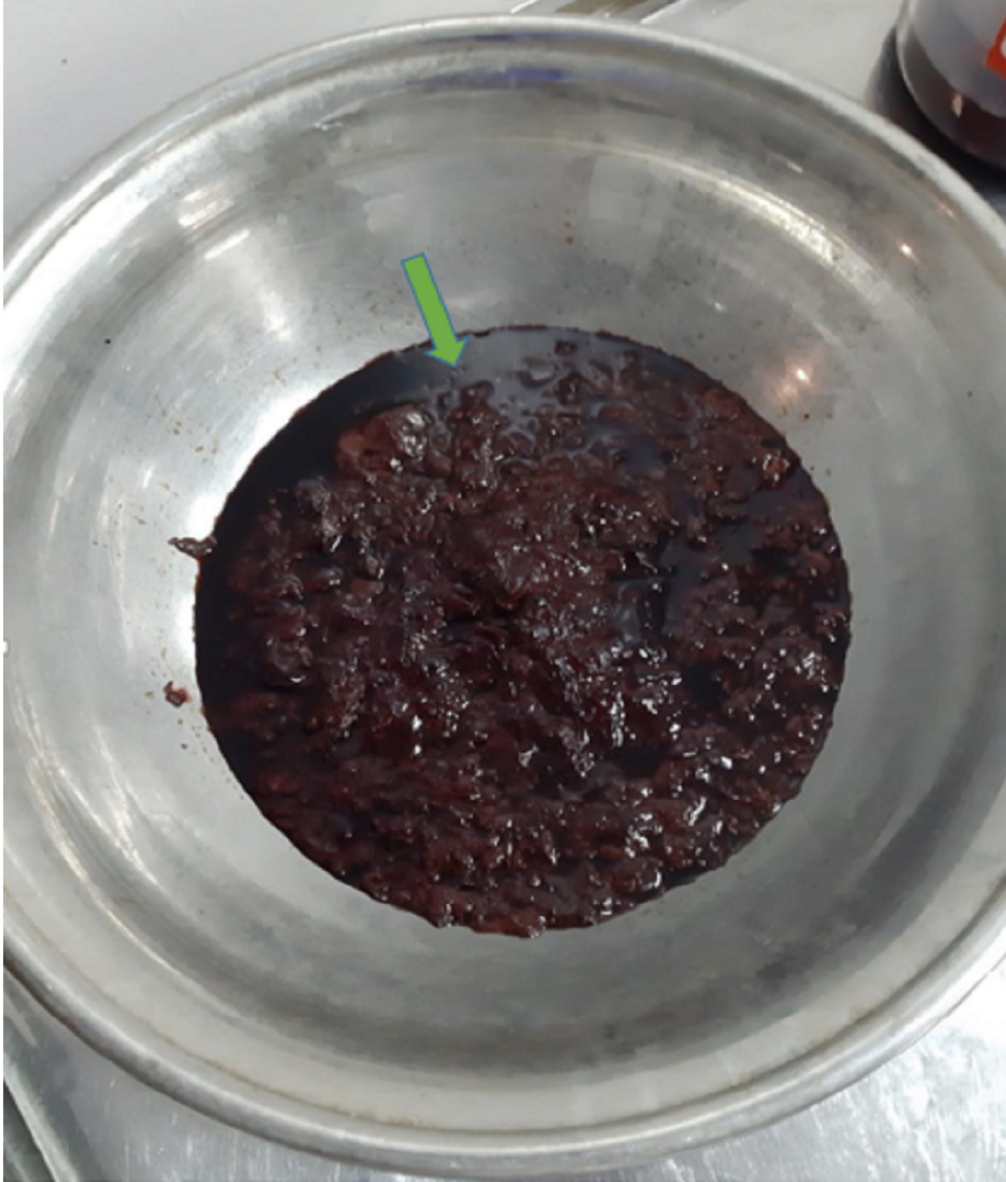
Collection of thick, dirty-colored material (green arrow) drained during operative procedure.

Surgery was conducted using a lateral approach. Once the incision was made, bloody, dark, dirty-colored thick material continuously drained out (Figures 3, 4). As a result, metallosis was suspected, and the collected material was sent for histopathology and detailed report (D/R).

**Figure 5:**
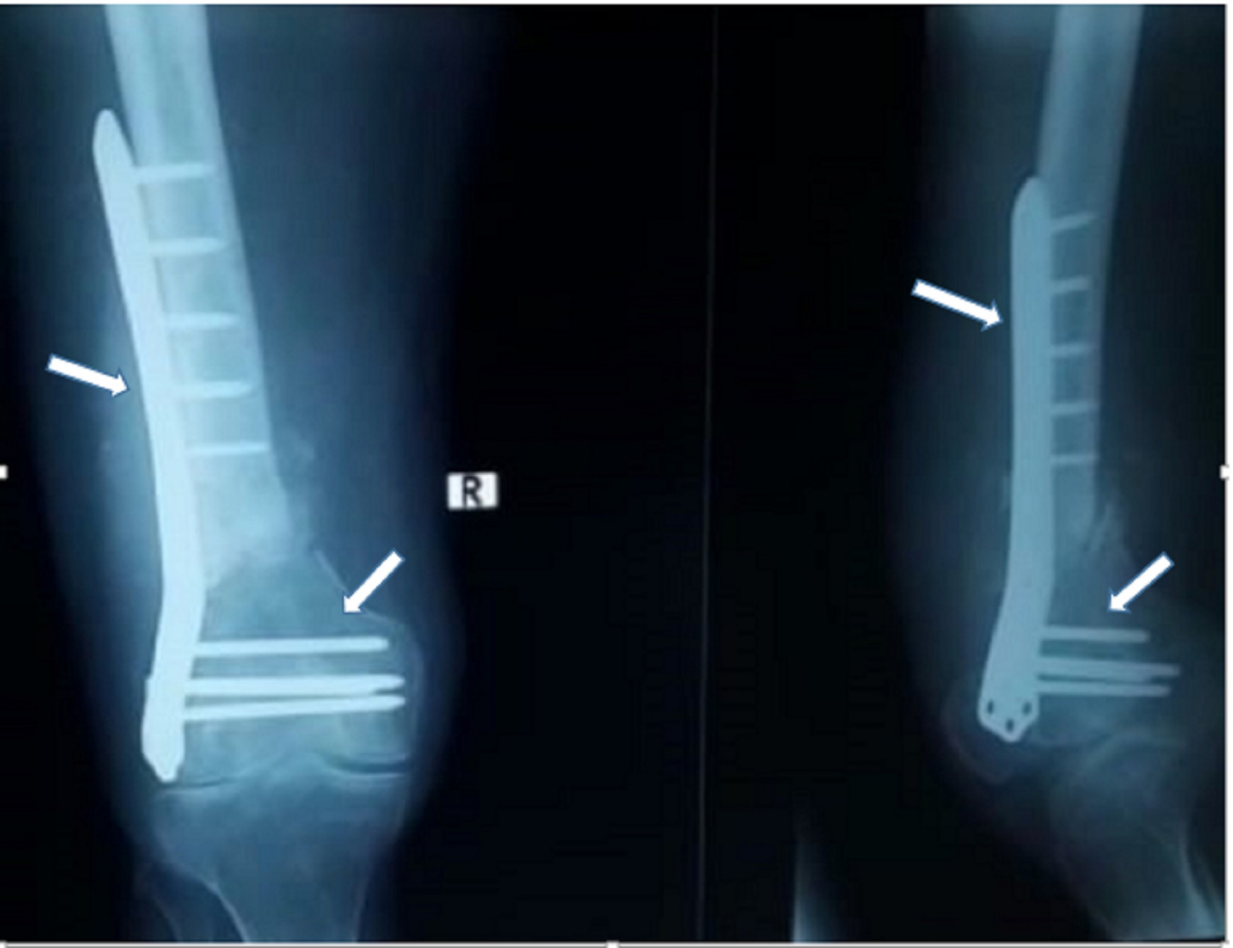
Lateral view (on right side of picture) and PA view (on left side of picture) X-rays of right distal femur showing well-reduced and aligned distal femur fracture after the surgical procedure with locking plate insertion (white arrows) and debridement of necrotic material. PA, posteroanterior

The fracture was identified, the broken plate was removed, and thorough wound debridement was completed. The fracture was then reduced and fixed using a distal femur locking plate and a bone graft. The wound was closed in layers, and the stitches were removed after two weeks. Gradual knee movements were allowed as tolerated. Follow-ups were conducted every two weeks, and the patient was allowed to bear partial weight after six weeks and full weight after 12 weeks. Subsequent X-rays were done to document the signs of the union, which showed sclerotic changes at the fracture site signifying primary healing, as the locking plate was inserted in compression mode (Figure 5). The patient was able to gain a full range of movement at four months' duration.

The histopathology report revealed multiple irregular brown to tan-colored tissue fragments collectively measuring 8 × 6 × 3 cm in dimension. One of the fragments showed hemorrhage and necrotic areas. Sections showed multiple fragments of fibrocollagenous, adipose, and muscle with effaced architecture by areas of hemorrhage congestion and hemosiderin-laden macrophages. Most of the tissue showed granulation, proliferated blood vessels, and scattered mixed inflammatory infiltration comprised of neutrophils, lymphocytes, and plasma cells. The larger blood vessels were thrombosed with fibrinous necrosis in some of the foci. A section from larger pieces showed necrotic bone spicules with fibroblastic proliferation and multinucleated giant cells. Although no specific findings pertinent to metallosis were documented by the pathologist, the clinical correlation and the absence of other etiologies were the findings enough to support the diagnosis of metallosis.

## Discussion

Metallosis usually poses a diagnostic challenge, and, on most occasions, even experts in the field tend to misdiagnose it. X-rays do not directly show metallosis, but the following two typical signs can provide clues that it is present: metal line sign (radiopacity due to metallic debris) and bubble sign (metallic debris outline joint surface). Metallosis is a slowly evolving process that takes months to years to develop and present clinically [6]. Although CT and MRI have an edge over X-ray in defining the soft tissues, due to financial constraints and contradiction to MRI (metal prosthesis in place), these imaging modalities were not used in our patient.

Clinically, metallosis can present with extreme swelling that mimics tumors, disabling pain, limb-length discrepancy, and inability to bear weight. All these symptoms were present in the reported patient. It is key to differentiate metallosis from infection. Low titers of infectious markers like erythrocyte sedimentation rate (ESR), C-reactive protein (CRP), and leukocytosis and radiographic signs supporting metallosis are usually enough to make a preliminary diagnosis of metallosis [7]. Local responses to metals consist of a wide clinical spectrum ranging from small asymptomatic tissue lesions to severe destruction of bone and soft tissues, which are designated as metallosis, adverse reactions to metal debris (ARMD), aseptic lymphocytic vasculitis-associated lesion (ALVAL), and pseudotumors [8]. 

Metallosis can elicit a chronic inflammatory response, which is characterized by tissue infiltration by macrophages, lymphocytes, and multinucleated giant cells, necrosis, fibrosis that damages the surrounding tissue, and synovium [9]. Friction between bearing surfaces, corrosion of non-moving parts, and loosened components in failed prosthesis increases metallosis [10].

For patients, like the one discussed above, who present with non-union of a distal femur fracture along with breakage of the plate, an orthopedist should be mindful of metallosis as one of the potential complications (although very rare). Once the clinical picture of metallosis is suspected/confirmed, the ultimate goal is to prevent further harm and to conduct timely exploration of the affected limb, debridement of necrosed tissue/bone, and revision surgery. In our case, we proceeded with the fixation of the distal femur fracture with a locking plate along with debridement with positive results.

## Conclusions

Metallosis is a progressive process that takes a significant length of time after surgical implant placement to develop and manifest clinically. It is often difficult to diagnose owing to its rarity and non-specific clinical and radiological findings. A failure to detect such radical implant degeneration can lead to a nexus of devastating local and systemic complications, which require aggressive interventions and long hospital stays. The key to prevent these complications is early diagnosis.
